# Macrophage migration inhibitory factor-794 CATT microsatellite polymorphism and risk of tuberculosis: a meta-analysis

**DOI:** 10.1042/BSR20171626

**Published:** 2018-07-06

**Authors:** Mingbiao Ma, Lvyan Tao, Aihua Liu, Zhang Liang, Jiaru Yang, Yun Peng, Xiting Dai, Ruolan Bai, Zhenhua Ji, Miaomiao Jian, Fukai Bao

**Affiliations:** 1 Department of Microbiology and Immunology, Kunming Medical University, Kunming 650500, China; 2 Department of Biochemistry and Molecular Biology, Kunming Medical University, Kunming 650500, China; 3 Yunnan Province Key Laboratory for Tropical Infectious Diseases in Universities, Kunming Medical University, Kunming 650500, China; 4 The Institute for Tropical Medicine, Kunming Medical University, Kunming 650500, China; 5 Infection Section, Yunnan Demonstration Base of International Science and Technology Cooperation for Tropical Diseases, Kunming 650500, China; 6 Infection Section, Yunnan Province Integrative Innovation Center for Public Health, Diseases Prevention and Control, Kunming Medical University, Kunming 650500, China

**Keywords:** MIF, microsatellite polymorphism, meta-analysis, tuberculosis

## Abstract

Tuberculosis (TB) is a chronic infectious disease that has been threatening public health for many years. Several studies have shown the relationship between the macrophage migration inhibitory factor (MIF)-794 CATT (MIF-794 CATT) microsatellite polymorphism and susceptibility to TB. However, the results remain inconclusive. Therefore, we aim to find out the impact of MIF-794 CATT microsatellite polymorphism on risk of TB by a comprehensive meta-analysis. We conducted a systematic study search in PubMed, Embase, the Cochrane Library, and the China National Knowledge Infrastructure (CNKI) up to October 2017. Five studies involving 836 cases and 678 controls were included in the current meta-analysis. We calculated the pooled odds ratios (ORs) and corresponding 95% confidence intervals (CIs) to estimate the association between the MIF-794 CATT microsatellite polymorphism and risk of TB. The reliability of the results were evaluated with trial sequential analysis (TSA). The results suggested that the MIF-794 CATT microsatellite polymorphism was significantly associated with the susceptibility of TB in all comparisons for allele (7 + 8 compared with 5 + 6, OR = 1.56, 95% CI = 1.31–1.87, *P*<0.00001) and genotype (7/X + 8/X compared with 5/X + 6/X, OR = 1.81, 95% CI = 1.39–2.36, *P*<0.0001). Therefore, the meta-analysis indicated the MIF-794 allele CATT_7_ and CATT_8_ may be a risk factor to increase the susceptibility of TB, which was confirmed by TSA.

## Introduction

Tuberculosis (TB) is a chronic infectious disease caused by the bacillus *Mycobacterium tuberculosis (Mtb)* and threats to global human health seriously. According to a recent World Health Organization (WHO) report, there were an estimated 10.4 million new TB cases worldwide and nearly 1.4 million people died of TB in 2015. Although the number of TB deaths fell by 22% between 2000 and 2015, TB still remained one of the top ten causes of death worldwide in 2015 [1]. Thus, the prevention and control of TB is drawing more and more attention of the medical profession.

Many studies have shown that several factors were associated with the progression to infection of TB, which mainly include environmental factors, smoking, HIV infection, individual differences, and others [2–4]. Moreover, some studies have confirmed that genetic susceptibility genes also play an essential role in the development of TB, and several gene polymorphisms have been demonstrated as being associated with susceptibility to TB. Studies revealed the positive association of HLA-DRB1*150124, 25, and HLA-DQB1*0601 (a subtype of HLA-DQ1) with susceptibility to pulmonary TB [5,6]. Several studies confirmed the crucial roles of TLR2, TLR4, and TLR8 in the development of TB [7–11]. Additionally, *IRGM1* (also known as *LRG47*) is the main autophagy gene that has been examined in TB patients, and different polymorphisms in the *IRGM1* gene were found to be associated with susceptibility to TB in various regions such as China, West Africa, and U.S.A. [12–14].

The gene of human migration inhibitory factor (MIF) is located on human chromosome 22q11.2. The MIF protein functions as a cytokine that promotes inflammatory processes in response to pathogenic infection. Studies have demonstrated that MIF can inhibit the migration and promote the aggregation of macrophages at sites of local inflammation or infection [15–17]. The two polymorphisms in the promoter region of MIF have been extensively identified. One is a single nucleotide polymorphism (SNP) at the nucleotide position −173 (G to C) and another is microsatellite polymorphism-794 CATT5-8 [18]. Some studies have suggested that the two polymorphisms of MIF closely related to susceptibility to TB [19–24], and several meta-analysis have conducted to regard the association between the MIF-173 G>C polymorphism and the susceptibility to TB [25,26]**.** However, there has not been a meta-analysis to evaluate the association between the MIF-794 CATT microsatellite polymorphism and risk of TB. Therefore, the present study aimed to perform a meta-analysis of all eligible studies to precisely investigate the association between the MIF-794 CATT microsatellite polymorphism and risk of TB.

## Methods

### Study selection

A comprehensive literature search of the PubMed, Embase, the Cochrane Library, and the China National Knowledge Infrastructure (CNKI) was conducted for articles published up to October 2017. The key search words were (‘macrophage migration inhibitory factor’ or ‘MIF’) and (‘polymorphism’ or ‘variant’ or ‘mutation’ or ‘genotype’) and TB. The language was restricted to English and Chinese.

The inclusion criteria were as follows: (i) the design should be a case–control study; (ii) the study should refer to the association between the MIF-794 CATT microsatellite polymorphism and risk of TB; (iii) distribution of alleles, genotypes, or other necessary information should be provided to calculate the odds ratio (OR) and 95% confidence interval (CI); (iv) the object of study should be human; (v) the study was written in English or Chinese. The exclusion criteria were as follows: (i) not designed as a case–control study; (ii) review, abstract, comment, or case report; (iii) insufficient data and information in article; (iv) not refer to the relationship between the MIF-794 CATT microsatellite polymorphism and risk of TB

### Quality assessment

We evaluated the qualities of included studies according to the Newcastle–Ottawa scale (case–control study), which was based on three aspects including selection, comparability, and exposure in the study. The total scores ranged from 0 to 9. All the included studies were assessed by two of our authors independently. Any disagreement was resolved by discussion with a third author. Each item should be able to reach a final consensus. Studies with a score of 7 or greater were considered to be of high quality.

### Data extraction

Extraction of data was carried out by two independent authors (M.M. and L.T.) according to the inclusive criteria. If there was a disagreement, the third author (Y.P.) assessed those articles. The data extracted included the first author’s name, date of publication, country, ethnicity of participants, genotyping methods, age, Hardy–Weinberg equilibrium (HWE) test, sample size, and the quality score of each study.

### Statistical analysis

The present meta-analysis was performed with the RevMan 5.0 and software Stata 12.0. We measured the strength of association between the MIF-794 CATT microsatellite polymorphism and risk of TB by the OR and 95% CI. The Chi-square test was used to assess the HWE in order to analyze the genotype distribution in the control groups. *Q*-test and *I^2^*test were used to evaluate the statistical heterogeneity amongst studies, *P*<0.05 or *I*^2^> 50% was considered to indicate statistically significant heterogeneity. If we observed the heterogeneity amongst studies, the pooled OR was calculated by random-effect models, otherwise, the fixed-effect model was applied.

### Trial sequential analysis

The small number of trials and patients would increase the random error and might cause spurious results. Therefore, we used the trial sequential analysis (TSA) to evaluate the reliability of the result for meta-analysis, and the TSA version 0.9.5.10 β was downloaded from the website (www.ctu.dk/tsa). The required information size (RIS) was calculated by *a priori* diversity-adjusted information size (APDIS) defining 20% for relative risk reduction (RRR), 5% for type I error (two-sided), and 80% for the power. TSA boundaries were constructed to assess the risk of random error when the number of available participants was less than the RIS. If a Z-curve crossed the futility boundaries or TSA boundaries, there was sufficient information to support the conclusions and further trials were unlikely to change the findings.

## Results

### Study characteristics

**Figure 1:**
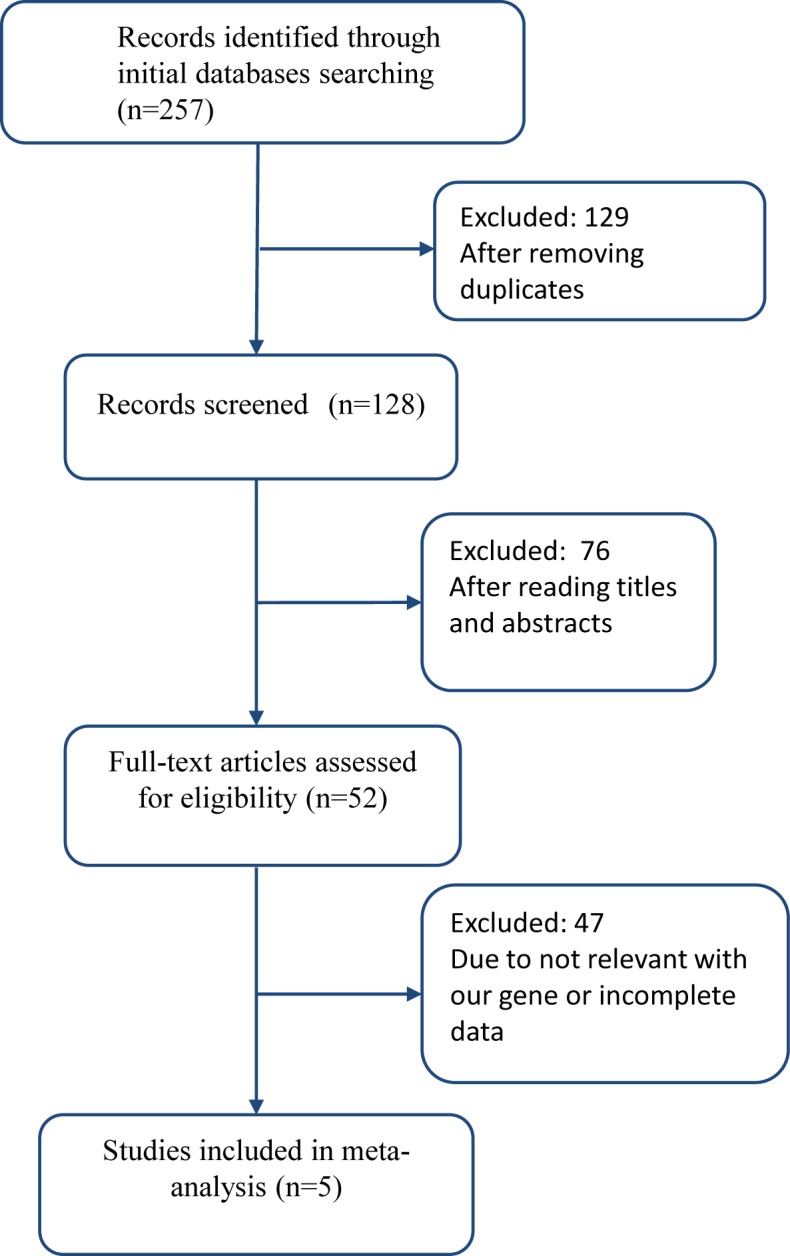
The flow diagram of the study selection in this meta-analysis

**Figure 2:**
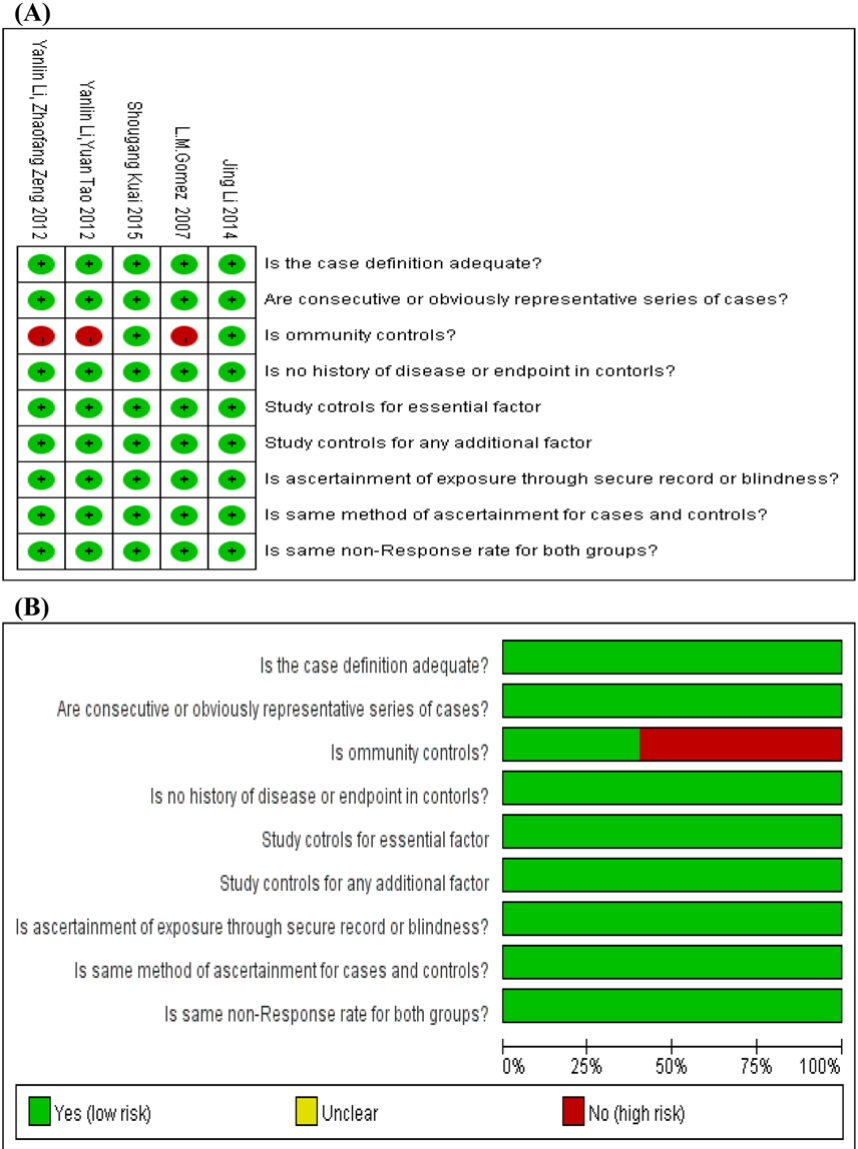
The details of quality assessment for each included study according to the Newcastle–Ottawa scale (**A**) The details about each risk of bias item for each included study. The red and green mean high risk and low risk, respectively for each included study. (**B**) The summary of each risk of bias item are presented as percentages across all included studies. The red and green mean high risk and low risk, respectively for each risk of bias item.

After a systematic search, 257 potentially relevant studies were yielded. After we removed 129 duplicates, we further read the titles and abstracts of the rest of studies, 76 studies did not meet our article’s theme apparently since they were review, comment, report, or did not refer to the MIF-794 CATT microsatellite polymorphisms and risk of TB. Then, we read full articles of remaining studies. Forty-seven of them were excluded because they were not connected with the polymorphisms of MIF gene or did not provide essential dates. Finally, five studies met the inclusion criteria and were included in the current meta-analysis. Figure 1 shows the details of selection process. In all the five studies, four studies were in English and one study was in Chinese. Additionally, the five studies included in the meta-analysis were of high quality according to the Newcastle–Ottawa scale, which indicated the methodological quality was generally good. The details about each risk of bias item for each included study were shown in Figure 2A, and the summary of each risk of bias item presented as percentages across all the eligible studies were listed in Figure 2B. The main characteristics of all the included studies were presented in Table 1, and the distribution of alleles and genotypes of MIF-794 CATT included in studies were provided in Table 2.

**Table 1:** Main characteristics of included studies in this meta-analysis

Author	Year	Country	Ethnicity	Genotyping methods	Mean age (year ± median)		Sample size		HWE test	Quality score
					Cases	Controls	Cases	Controls		
Jing Li [24]	2014	China	Asian	Taqman	40.34 ± 17.85	28.65 ± 9.15	200	100	Y	9
L.M. Gomez [19]	2007	Colombia	Latin American	Taqman	40 ± 16	43 ± 16	223	134	Y	8
Shou-Gang Kuai [20]	2015	China	Asian	Taqman	48.4 ± 10.5	48.4 ± 10.5	47	50	Y	9
Yanlin Li and Yuan Tao [21]	2012	China	Asian	Taqman	43.8 ± 5.76	40.2 ± 4.9	215	245	Y	8
Yanlin Li and Zhaofang Zeng [22]	2012	China	Asian	Taqman	47.8 ± 5.76	40.2 ± 4.9	151	149	Y	8

Abbreviation: Y, Yes.

**Table 2:** The distribution of alleles and genotypes of MIF-794 CATT in included studies in this meta-analysis

Author	Year			Genotypes				Alleles			
		Sample size		Cases		Controls		Cases		Controls	
		Cases	Controls	5/X + 6/X	7/X + 8/X	5/X + 6/X	7/X + 8/X	5 + 6	7 + 8	5 + 6	7 + 8
Jing Li	2014	200	100	136	64	77	23	315	85	175	25
L.M. Gomez	2007	223	134	NA	NA	NA	NA	368	78	223	46
Shou-Gang Kuai	2015	47	50	21	26	36	14	NA	NA	NA	NA
Yanlin Li and Yuan Tao	2012	215	245	93	122	134	111	266	164	363	127
Yanlin Li and Zhaofang Zeng	2012	151	149	124	27	137	12	195	107	222	76

5 + 6, the MIF-794 CATT alleles (5 + 6); 7 + 8, the MIF-794 CATT alleles (7 + 8); 5/X + 6/X, the MIF-794CATT genotypes (5/X + 6/X); 7/X + 8/X, the MIF-794 CATT genotypes (7/X + 8/X). Abbreviation: NA, not available.

### Meta-analysis

**Figure 3:**
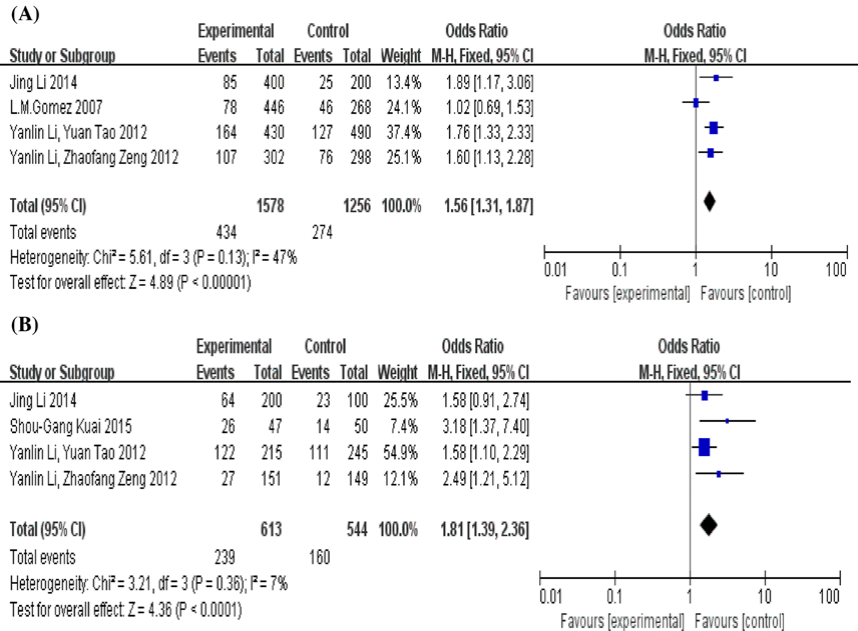
Forest plot of the association between the MIF-794 CATT microsatellite polymorphism and risk of TB (**A**) Alleles (7 + 8 compared with 5 + 6), (**B**) genotypes (7/X + 8/X compared with 5/X + 6/X). The squares and horizontal lines correspond to the study-specific OR and 95% CI, respectively. The area of the squares reflects the study-specific weight. The diamond represents the pooled results of OR and 95% CI. Overall analysis using the fixed-effect model. Abbreviations: fixed, the fixed-effect model; M–H, the method of Mantel–Haenszel.

In total, five studies involving 836 cases and 678 controls were included in our meta-analysis. As we can see from Figure 3, the results of *Q*-test and *I^2^* test showed *P*>0.1 and *I^2^* < 50%, which means there was no heterogeneity amongst the included studies both in comparison for allele (5 + 6 compared with 7 + 8) and genotype (5/X + 6/X compared with 7/X + 8/X). Therefore, we used the fixed-effect model to investigate the pooled OR. The results of both indicated that there are significant associations between the MIF-794 CATT polymorphism and risk of TB in allele (7 + 8 compared with 5 + 6, OR = 1.56, 95% CI = 1.3–11.87, *P*<0.00001) and genotype (7/X + 8/X compared with 5/X + 6/X, OR = 1.81, 95% CI = 1.39–2.36, *P*<0.0001). So, the MIF-794 allele CATT**_7_** and CATT**_8_** may be the factors in increasing the risk of TB infection.

### Publication bias

**Figure 4:**
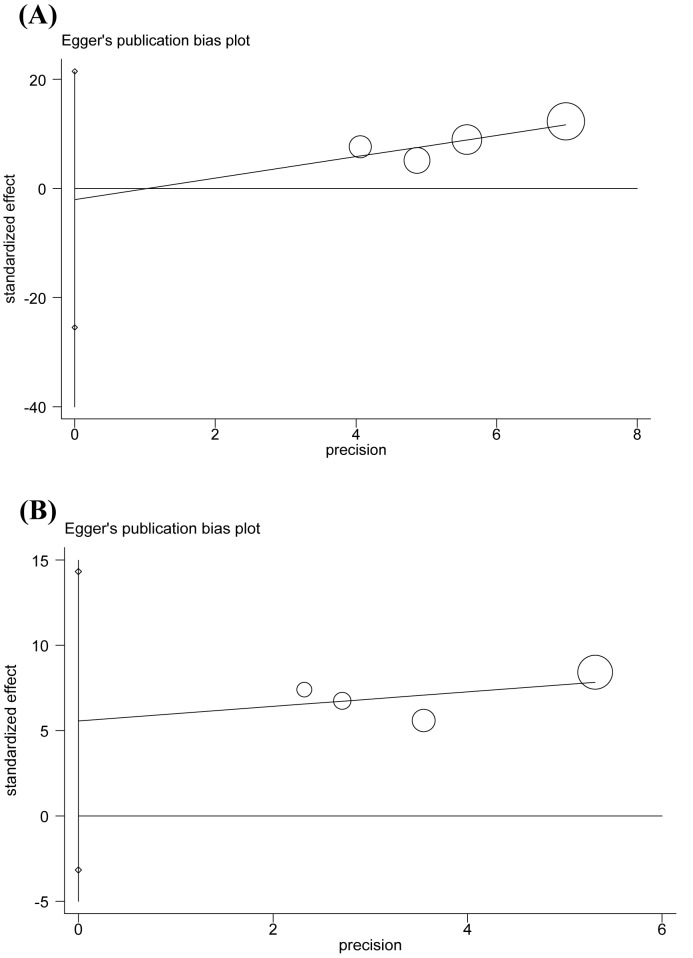
Funnel plot for evaluating publication bias on the association between the MIF-794 CATT microsatellite polymorphism and risk of TB (**A**) Egger’s test of the MIF-794 CATT alleles (7 + 8 compared with 5 + 6), (**B**) Egger’s test of the MIF-794 CATT genotypes (7/X + 8/X compared with 5/X + 6/X).

We carried out Begg’s funnel plot and Egger’s test to evaluate the publication bias of the included studies. The results are presented in Table 3 and Figure 4. No evidence of publication bias was observed (*P*=0.734/0.749 for allele, *P*=0.089/0.111 for genotype).

**Table 3:** A summary of *P*-values for Begg’s funnel plot and Egger’s test in allele and genotype

MIF-794 CATT	Begg’s funnel plot	Egger’s test
Allele	0.743	0.749
Genotype	0.089	0.111

### Sensitivity analysis

We performed sensitivity analysis to evaluate the reliability of the meta-analysis by deleting one single study each time. Although the sample size is a little less in our meta-analysis, however, the results both showed the stability of the study was not influenced by any individual study in allele and genotype. The results of sensitivity analysis were presented in Table 4.

**Table 4:** The results of sensitivity analysis for allele and genotype

Study omitted	e^coef	95% CI	
**Allele**			
Jing Li (2014)	4.7195315	3.8928899	5.7217076
L.M. Gomez (2007)	5.6544964	4.6313477	6.9036771
Yanlin Li and Yuan Tao (2012)	4.9347892	4.0089238	6.0744843
Yanlin Li and Zhaofang Zeng (2012)	4.4172572	3.5021082	5.5715472
Combined	4.9431385	4.133599	5.9112211
**Genotype**			
Jing Li (2014)	1.9564271	1.6503177	2.2625365
Shou-Gang Kuai (2015)	1.7194507	1.4371029	2.0017985
Yanlin Li and Yuan Tao (2012)	2.182389	1.7931269	2.5716511
Yanlin Li and Zhaofang Zeng (2012)	1.7682616	1.479958	2.0565651
Combined	1.8669294	1.5991781	2.1346807

### TSA

**Figure 5:**
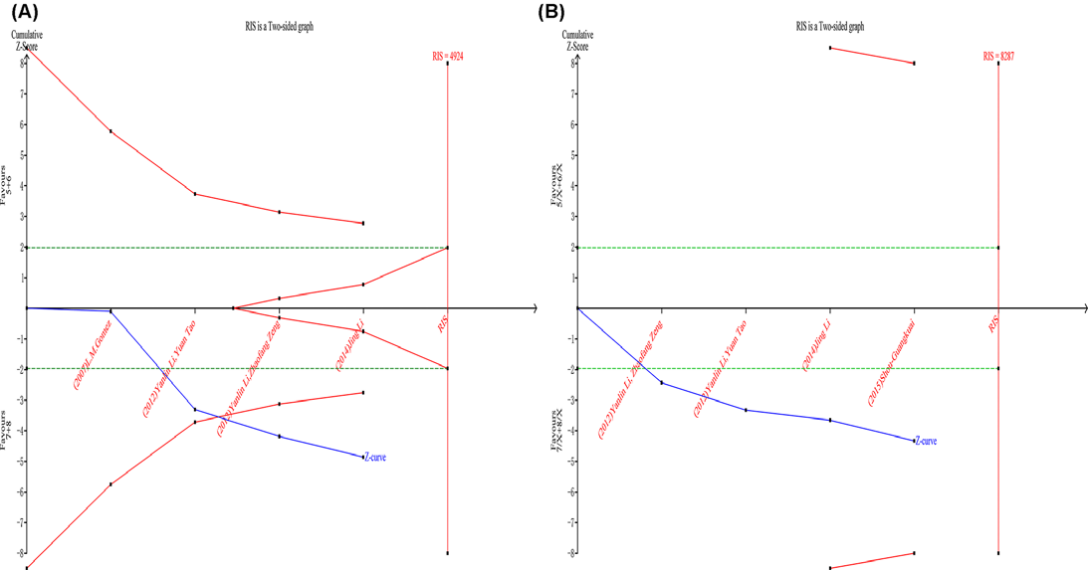
The results of TSA for the MIF-794CATT alleles and genotypes (**A**) Alleles, (**B**) genotypes. The blue solid line represents the cumulative Z-curve. The horizontal green dotted lines represent the conventional boundaries. The red lines represent the TSA boundaries. The red area arising at the right of the horizontal axis represents the futility area.

TSA was performed as described by user manual to analyze whether the available data were powered enough to reach firm conclusions in the present study. For allele, TSA showed the RIS was 4924 patients and the Z-curve crossed both the conventional boundary and TSA boundary, which indicated that although the current trials did not reach the RIS, the result was conclusive and further trials were unlikely to change the conclusion (Figure 5A). For genotype, RIS was 8287 patients and the Z-curve only crossed the conventional boundary, which means more trials were needed to confirm conclusion (Figure 5B).

## Discussion

Macrophage MIF was originally discovered at the process of study on delayed-type hypersensitivity by Boom and David in 1966, which was named for inhibiting the migration of macrophages. MIF was mainly expressed in macrophages, and their cell types can also produce products such as T/B lymphocytes, monocytes, and dendritic cells [27]. MIF plays an important role in the pathogenesis of immune diseases by inhibiting the migration of macrophage, and promote the aggregation, invasion, proliferation, activation, and secretion of several cytokines of the macrophage at the site of infection to produce a strong immune response. At the moment, the MIF can also promote the release of NO and cyclooxygenase (COX-2) to interact with inflammatory cytokines, which can enhance the inflammation injury [28]. Thus, MIF plays an important role in the incidence and development of TB. The expression of MIF was regulated by the MIF promoter polymorphism.

Human MIF gene is a single copy gene including three exons and two introns. Currently, four polymorphisms have been identified in MIF gene: a microsatellite polymorphism at position −794 (CATT5-8) and three SNPs at positions −173 (rs755622), +254 (rs2096522), and +656 (rs2070766). However, because +254 (rs2096522) and +656 (rs2070766) are located in the intron, so only the MIF-173 (rs755622) and MIF (CATT_5–8_) have been reported to affect the MIF level. The MIF-173C allele has been associated with a higher production of MIF protein [18], and the MIF-794 CATT repeats have been found to be associated with levels of MIF gene transcription *in vitro* [29]. Many studies also mainly focus on researching the association between the two MIF polymorphisms and diseases. For example, Liu et al. [30] revealed that MIF-173 polymorphism may be a risk factor for new-onset Graves’ disease (GD) in Taiwanese Chinese population. Compared with GD patients carrying G/G genotype at the MIF-173 SNP, those carrying G/C or C/C genotypes were with higher free thyroxine (FT4) level. Li et al. [22] found that MIF-794 microsatellite polymorphism may be a risk factor to increase the susceptibility of TB. The MIF-794 CATT_5–8_ microsatellite polymorphism is associated with the alteration of MIF gene transcription levels. The repeat number of the CATT can regulate the activity of MIF gene promoter. The higher the repeat number, the stronger the activity of the promoter.

This is the first meta-analysis to investigate the association between the MIF-794 CATT microsatellite polymorphism and risk of TB, our meta-analysis indicated the MIF-794 microsatellite polymorphism may be associated with the susceptibility of TB. The MIF-794 allele CATT**_7_** and CATT**_8_** may be the factors in increasing the risk of TB infection.

There were several limitations in the present meta-analysis. First, due to lack of sufficient data and studies, we failed to perform further subgroup meta-analysis to evaluate the TB risk factors such as ethnicity, age, gender etc., which may play a profound role in the infection of TB. These factors had an effect on the heterogeneity in meta-analysis. Second, we only included studies published in English and Chinese, it is possible that some pertinent studies published in other languages may have been missed. At last, most of the included studies were conducted in Asian population, whereas other population should be included in the future analysis. Despite these limitations, we minimized the likelihood of bias through the whole process by creating a detailed protocol, by performing study identification, data selection, and statistical analysis, as well as in the control of publication bias.

In summary, the present meta-analysis suggests that there are significant association between the MIF-794CATT polymorphism and risk of TB, and allele CATT**_7_** and CATT**_8_** be the factors in increasing the risk of TB infection. For genotype CATT_7/X + 8/X_, there is a need to perform with well-designed case–control studies and larger sample size focussing on more ethnicities to confirm the results in the future.

## Abbreviations

CATT: microsatellite polymorphism of cytosine deoxyribonucleotide, adenyl deoxyribonucleotide,thymine deoxyribonucleotide, and thymine deoxyribonucleotide
CI: confidence interval
COX-2: cyclooxygenase-2
FT4: free thyroxine
GD: Graves’ disease
HWE: Hardy–Weinberg equilibrium
MIF: migration inhibitory factor
OR: odds ratio
RIS: required information size
SNP: single nucleotide polymorphism
TB: tuberculosis
TSA: trial sequential analysis
TLR: Toll-like receptor
